# Renal Biopsy for Diagnosis in Kidney Disease: Indication, Technique, and Safety

**DOI:** 10.3390/jcm12196424

**Published:** 2023-10-09

**Authors:** Peter Schnuelle

**Affiliations:** Center for Renal Diseases Weinheim, Academic Teaching Practice of the University Medical Center Mannheim, University of Heidelberg, D-69469 Weinheim, Germany; p.schnuelle@nierenzentrum-weinheim.de; Tel.: +49-6201-947931; Fax: +49-6201-947920

**Keywords:** renal biopsy, indication, clotting disorder, hypertension, real-time ultrasound, spring-loaded biopsy device, biopsy needle size, bleeding, laparoscopic-assisted biopsy, transjugular renal biopsy

## Abstract

Renal biopsies are the gold standard for diagnosis, staging, and prognosis of underlying parenchymal kidney disease. This article provides an overview of the current indications and highlights ways to reduce bleeding complications in order to achieve optimal diagnostic yield with minimal risk to the patient. Novel indications have emerged from the increasing use of new molecularly targeted oncologic therapies in recent years, which often induce immune-mediated renal disease. On the other hand, the detection of specific antibodies against target antigens on podocytes in the sera of patients with new-onset nephrotic syndrome has now relativized the indication for biopsy in membranous nephropathy. The use of semi-automatic spring-loaded biopsy devices and real-time ultrasound considerably declined the complication rate and is the current standard. Percutaneous renal biopsies are overall a safe procedure if contraindications are considered. A coagulation disorder needs to be excluded beforehand, and an elevated blood pressure must be reduced to the normotensive range with medications. A laparoscopic approach or a radiology interventional procedure through the internal jugular vein may be considered for obtaining a kidney tissue sample if there is an urgent indication and a bleeding tendency cannot be adequately corrected. Major bleeding after a percutaneous renal biopsy can usually be managed with selective arterial embolization of the injured renal vessel. The use of a 16-gauge needle is the most reasonable compromise between diagnostic benefit and risk of complication. In the routine diagnostic, the biopsy specimen is examined with light microscopy, immunohistochemistry, and electron microscopy. Combination with modern molecular pathology techniques will contribute to more precise insights into the development and progression of kidney disease, which will likely refine future treatments in nephrology.

## 1. Introduction

Acute and progressive chronic kidney diseases are subject to a variety of inflammatory and autoimmune processes, which are often accompanied by degenerative lesions or are also genetically determined. In many cases, the underlying causes cannot be distinguished clinically, nor can they be identified by advanced laboratory tests because they remain confined to the renal parenchyma. Therefore, a renal biopsy is indicated when knowledge of the histological diagnosis is essential for appropriate therapy [1,2]. Nevertheless, there is general agreement that the biopsy findings should always be viewed and interpreted in the context of clinical and historical data. In addition to histological diagnosis, a renal biopsy also allows the prognosis of underlying renal disease to be assessed. However, the advantages of histological diagnosis must always be weighed against the possible risks due to the invasive nature of the procedure [3,4]. For optimization of the diagnostic significance, the biopsy sample should routinely be subjected to light microscopy, immunofluorescence, and electron microscopy [5,6,7]. Additional new modern pathology techniques based on gene expression analysis and proteomics, in situ detection of functionally relevant molecules, and new bioinformatics approaches have the potential to provide deep insights into the mechanisms behind the morphological findings and refine molecular pathways for treatment interventions [8,9]. A recent example of this is severe acute respiratory syndrome coronavirus type 2 (SARS-CoV-2) associated nephropathy. SARS-CoV-2-associated nephropathy, in particular, has been extensively characterized through the application of state-of-the-art molecular pathological methods. Both the receptor protein (angiotensin-converting enzyme 2 [ACE-2] receptor) and the virus itself have been visualized in tubular cells using fluorescence in situ hybridization [10,11]. Acute tubular injury is the most common renal involvement in patients infected with SARS-CoV-2, followed by thrombotic microangiopathy (TMA) and necrotizing glomerulonephritis, establishing SARS-CoV-2-nephropathy as a distinct entity.

The available evidence suggests that the histological diagnosis of both native and transplanted kidney biopsies has a direct therapeutic impact or significantly influences the patient’s further treatment in about 40–60% of cases [12,13]. Apart from protocol biopsies for scientific purposes, the indication for performing a renal biopsy is determined in individual cases by the subjective assessment of the treating nephrologist with regard to the therapeutic benefit [14,15]. There are considerable differences in the indication and performance of a kidney biopsy locally in the nephrology departments and also in an international comparison [16,17]. Comparing the biopsy frequency in Australia and in the USA on the basis of a cross-sectional survey from 1995 to 1997 revealed that more than 250 kidney biopsies per million population were performed in Australia, while this figure was less than 75 per million population in the USA [18].

## 2. Indications

Table 1 summarizes the current indications for native and transplant kidney biopsies. Classic indications for biopsy of the patient’s own kidney include new-onset nephrotic syndrome in adults, evidence of proteinuria greater than 1–2 g/24 h with or without hypertension, and impaired renal function of unknown cause, especially when an active urine sediment indicates possible crescentic glomerulonephritis. Furthermore, if renal involvement is suspected in association with an immunological or paraneoplastic systemic disease, e.g., antineutrophil cytoplasmatic antibody (ANCA)-associated vasculitis, systemic lupus erythematosus (SLE), monoclonal gammopathy, or amyloidosis [1,2]. In the case of isolated microscopic hematuria without renal function impairment, a biopsy is only indicated in exceptional cases (e.g., for clarification before potential living donation), as there is usually no therapeutic consequence due to an overall favorable prognosis [1]. However, the kidney biopsy renders it possible to distinguish the underlying entities. The most frequent differential diagnoses include immunoglobin A (IgA) nephropathy, hereditary nephritis (classic Alport syndrome), and thin basement membrane syndrome, which, according to recent findings, is attributed to the autosomal recessive form of Alport syndrome in the case of heterozygosity [19,20,21]. In instances of mild proteinuria below 1 g/24 h without an active sediment and without clinical or serological evidence of a systemic disease, many nephrologists relativize the indication for renal biopsy [1,2]. Immunosuppressive therapy is usually not indicated. However, these patients require regular monitoring. If proteinuria increases, hypertension occurs and/or renal function decreases, a histological clarification should be sought [2]. Biopsy of nephrotic syndrome in childhood is usually not necessary. In more than 90% of cases, glomerular minimal lesions are present that respond very well to steroids. However, in steroid-resistant nephrotic syndrome, renal biopsy is indicated and can be performed safely [22,23].

In adults, membranous nephropathy (MN) and focal segmental glomerulosclerosis are among the most common causes of nephrotic syndrome, ahead of minimal glomerular lesions. Unexpected diagnoses, such as primary amyloidosis, fibrillary or immunotactoid glomerulopathy, and rare diseases, such as Fabry disease, expand the differential diagnostic spectrum with the resulting therapeutic implications. In recent years, antibodies to target antigens on podocytes underlying primary MN have been identified, namely antibodies to phospholipase A2 receptor (PLA2R-Abs) and, less frequently, to thrombospondin type-1 domain-containing protein 7A (THSD7A-Abs) [24,25]. Although it is clear that a kidney biopsy is required in the absence of PLA2R-Abs, their detection in serum is so specific that histological clarification in new-onset nephrotic syndrome is not necessary. Studies have reliably shown that a biopsy at an eGFR of >60 mL/min does not provide an additional diagnosis that would change clinical management, nor does it provide additional information about chronicity or prognosis [26].

Acute nephritic syndrome, clinically characterized by hematuria with microscopic evidence of acanthocytes and/or red blood cell casts, new-onset arterial hypertension, and renal insufficiency, definitely requires biopsy clarification to establish the diagnosis and further therapy [1,2]. It occurs isolated to the kidneys but also in association with immunological systemic disease (e.g., SLE, ANCA-associated vasculitis, and rarely anti-glomerular basal membrane (anti-GBM) nephritis). In ANCA-associated vasculitis, renal histology provides important information on whether the disease is active. Particularly in myeloperoxidase (MPO)-positive disease when confined to the kidneys without other systemic disease symptoms, a biopsy is essential for the indication of immunosuppressive therapy and also for renal prognosis, as it cannot be reliably estimated from conventional laboratory data of sediment, serum creatinine, and autoimmune serology. It has been shown that the number of unaffected normal glomeruli in a representative biopsy not only predicts response to therapy but also provides a prognosis for recovery of renal function after one year [27].

In lupus nephritis, immunosuppressive therapy depends largely on the pattern of involvement and the extent of active or chronic (scarred) lesions [28,29], which cannot be differentiated clinically. Repeated kidney biopsies may also be indicated, as the histological classification criteria overlap considerably and, due to the relapsing course of the disease under immunosuppressive therapy, non-proliferative forms of nephritis often change into proliferative forms and vice versa. Thus, individual patients can pass through different lupus nephritis classes in the course of their disease, with corresponding therapeutic implications [30].

Acute renal failure (prerenal renal failure and acute tubular necrosis) usually does not require a biopsy as long as a typical history and clinical circumstances make the diagnosis plausible [2]. On the other hand, a biopsy should be sought in the case of otherwise unexplained, newly occurring renal function impairment, especially for the etiological clarification of drug toxic causes [2]. In instances of suspected drug-induced acute interstitial nephritis (AIN), the identification of the culprit substance is not always easy because of the broad spectrum of possible substances. The injurious immunologic reaction in AIN is a cell-mediated process that usually manifests 7 to 10 days after exposure to the culprit substance [31]. Thus, clinical workup of AIN requires an accurate history of concomitant medication, including self-medications, the determination of the period between exposure and onset as accurately as possible, and the exclusion of other autoimmune or infectious diseases [32]. Frequent causative substances are analgesics of the non-steroidal anti-inflammatory drug (NSAID) type, which are usually available over the counter, but also several antibiotics and proton pump inhibitors (PPIs) [33]. It is necessary to identify the causative substance as precisely as possible because affected patients have to avoid it for the rest of their lives.

In more recent times, several indications for doing a renal biopsy have evolved from the use of novel tumor therapies in modern oncology. These therapies include tyrosine kinase inhibitors, which quite often confer direct nephrotoxic effects [34,35], but also monoclonal antibodies or recombinant fusion proteins directed against vascular endothelial growth factor (VEGF) or its receptor (bevacizumab and aflibercept), and checkpoint inhibitors [36,37]. Checkpoint inhibitors targeting immune proteins on T cells, such as programmed cell death 1 (PD-1) protein or its ligand (PDL-1), and cytotoxic T lymphocyte antigen 4 (CTLA-4), are frequently associated with various immune phenomena involving the kidneys, such as interstitial nephritis, TMA, and podocytopathy [36,38,39]. When renal function deteriorates or proteinuria occurs, with or without sediment findings, knowledge of renal histology can be helpful not only in characterizing the underlying cause but also in deciding on therapeutic measures to be taken to restore kidney function [39,40]. Sometimes, even effective tumor therapy must be interrupted or discontinued, and it may be necessary to switch to an alternative regimen and also consider systemic therapy with corticosteroids or immunosuppressants. It is not uncommon for a therapeutic dilemma to arise that requires close collaboration between oncologists and nephrologists.

Renal graft biopsy is the method of choice for the diagnosis and resulting treatments of various allograft injuries, including acute or chronic active rejection [41], BK polyomavirus-associated nephropathy [42], calcineurin inhibitor toxicity, and recurrent or de novo glomerular disease [43]. Due to the variety of mostly therapeutically modifiable disorders of graft function [44], biopsies of transplant kidneys (and re-biopsies) are performed more frequently than those of native kidneys. An indication in the early postoperative phase is generally given in cases of primary non-function, suspected acute rejection, or nonresponse to rejection therapy, but usually also in cases of deterioration of graft function in the further course or if proteinuria is detected above 1–2 g/24 h. Kidney allograft rejection has been graded according to the Banff Classification since 1991 [45], which has been revised and further developed over the years by an international expert panel of nephrologists, transplant surgeons, and pathologists at regular meetings. Key points of the most recent Banff 2019 classification mainly focused on the refinements to the criteria for chronic active T cell-mediated rejection (TCMR), borderline rejection, and antibody-mediated rejection (ABMR). In addition, the Banff Molecular Working Group (BMWG) elaborated a multiorgan gene panel, which is hoped to enable a pathogenesis and pathway-based molecular approach for diagnostics and therapeutic decision making [9,41].

**Table 1:** Indication for renal biopsy.

Biopsy of the native kidney to diagnose unknown renal disease
Adult nephrotic syndrome [1,2] Excepting new-onset nephrotic syndrome with evidence of PLA2-R-Abs [24,26]
Proteinuria > 1–2 g/24 h with or without hypertension [2] Excepting proteinuria/albuminuria associated with diabetes mellitus in the presence of proven diabetic retinopathy [2]
Progressive increase in serum creatinine with microscopic evidence of acanthocytes and/or red blood cell casts [1,2]
Systemic diseases (immunological or paraneoplastic) with suspected renal involvement [1,2] e.g., clinical/serologic evidence of systemic vasculitis c/p-ANCA positive and PR3-/MPO-Abs positive [1,2,27] e.g., clinical/serologic evidence of systemic lupus erythematosus (SLE) [1,2,30] e.g., serologic evidence of monoclonal gammopathy [1]
Impaired renal function of unclear etiology (if kidneys are of normal size on ultrasound) with or without sterile pyuria/white blood cell casts/low-grade proteinuria [1,2]
e.g., drug-induced interstitial nephritis [2,31,33]
e.g., interstitial nephritis related to autoimmune diseases (sarcoidosis, IgG4-related disease) [2]
Repeated biopsy to examine severity of damage or progression of an already-known kidney disease
Nonresponse to an established therapy [22,23] e.g., steroid resistance with glomerular minimal lesions
Therapy monitoring [1,2,30] e.g., clarification of whether immunosuppressive therapy needs to be intensified or can be suspended in individual cases of SLE or ANCA-associated vasculitis
Recurrent disease activity [1,2,27,30] e.g., evaluation of active/chronic (scarring) lesions prior to resumption of immunosuppressive therapy.
Biopsy of the renal graft
All the conditions of the native kidney
Special considerations for the kidney transplant
Primary non-function after transplantation Differentiation of acute tubular necrosis (ATN) from early rejection [41]
Rapid graft function impairment, in particular when rejection is suspected Grading according to Banff classification with regard to prognosis and treatment [41] Kind of rejection (cellular/antibody-mediated) [41] Differentiation of active/chronic lesions [41]
Clarification of nonresponse to rejection therapy Guidance of intensified immunosuppressive therapy [44] Detection/exclusion of infectious causes (BK-polyoma nephropathy) [42]
Deterioration of graft function in the ongoing course with or without proteinuria > 1–2 g/24 h Differentiation of chronic active rejection/late onset rejection/tubulointerstitial fibrosis (IFTA) [41] Diagnosis of recurrent kidney disease [43] Recognition of drug toxicities (calcineurin inhibitor toxicity) [41]
Control biopsies at predetermined time points (in context of clinical trials only)

Abbreviations: PLA2-R-Abs: phospholipase A2-receptor antibodies, c-ANCAs: antineutrophil cytoplasmatic antibodies, p-ANCAs: perinuclear antineutrophil cytoplasmatic antibodies, PR3-Abs: proteinase 3 antibodies, MPO-Abs: myeloperoxidase antibodies, SLE: systemic lupus erythematosus, and IgG4: immunoglobulin G4.

## 3. Contraindications

The most important contraindication to percutaneous kidney biopsy is a coagulation disorder. Therefore, global coagulation tests (platelet count, International Normalized Ratio (INR), Partial Thromboplastin Time (PTT), and Thrombin Clotting Time (TCT)) must be normal before doing a renal biopsy [46]. Higher-grade anemia also increases the incidence of bleeding complications and should be corrected beforehand [46,47]. In more severely impaired renal function, bleeding time is often prolonged due to impaired platelet aggregation. However, its significance with regard to the risk of complications after renal biopsy is controversial. Some observational studies indicate that complications occur at a 3–5-fold increased incidence with prolonged bleeding time [48,49]; other case series could not confirm this [50]. The administration of desmopressin may shorten the bleeding time in uremic patients with an urgent biopsy indication. Controlled clinical data on this issue are sparse. In a prospective, controlled, single-center study of 162 adult patients, desmopressin administration significantly reduced the size of perinephric hematomas. However, gross hematuria did not occur in any patient, and no change in hemoglobin level was noted after biopsy. Bleeding complications requiring therapeutic intervention were virtually absent in either study arm [51]. It, therefore, remains unclear whether serious bleeding complications can actually be prevented by desmopressin. A recent propensity score-adjusted retrospective study found higher odds for bleeding in patients who had received desmopressin (OR 3.88; 95% CI 1.95–7.74). Clearly, the study does not show causality with desmopressin administration, but it argues strongly against the ability of desmopressin to prevent severe bleeding complications [52].

Substances that inhibit platelet aggregation, such as acetylsalicylic acid (ASA), glycoprotein (GP) IIb/IIIa inhibitors, and NSAID, should be discontinued 7–10 days before elective renal biopsy according to current recommendations [1,2]. In the case of an urgent indication (e.g., progressive increase in serum creatinine with an active sediment or acute allograft rejection) or in a high-risk vascular situation, a biopsy can apparently be performed safely under ASA at a low dose of 100 mg [53,54]. A systematic meta-analysis on this topic found no association with serious bleeding complications in patients in whom ASA was continued, with the caveat that the overall quality of studies was considered low and prospective controlled clinical data were lacking [55]. In patients who have previously taken phenprocoumon or warfarin, the INR value must be normal. Similarly, direct oral factor Xa inhibitors must be paused 2–3 days beforehand [1,2]. The use of intravenous heparin as bridging anticoagulation in patients at high risk of thromboembolism must be discontinued approximately 6 h before the procedure and should not be resumed—if possible—until 12–24 h hereafter [1]. If medically justifiable, it is recommended to suspend full anticoagulation for at least 48–72 h after a percutaneous biopsy [1,2]; based on the author’s own experience, longer is preferable. Bleeding can still occur days after heparin therapy due to the onset of hyperfibrinolysis. If a coagulation disorder cannot be resolved, a surgical approach that allows hemostasis under vision or a transjugular renal biopsy (TJRB) should be considered.

Hypertension has previously been reported as a risk factor for biopsy complications [3,56,57]. However, according to more recent data, the presence of hypertension alone, if controlled by medication, does not seem to be associated with an increased risk [58]. This certainly also reflects today’s generally more cautious indication for performing a percutaneous kidney biopsy if blood pressure is not well controlled. An uncontrolled blood pressure exceeding 140/90 mmHg is considered a contraindication to percutaneous renal biopsy [59]. Patients in whom blood pressure cannot be adequately lowered and who urgently require a renal biopsy may benefit from a surgical or an interventional procedure [2].

Another clear contraindication to percutaneous renal biopsy is the patient’s inability to follow instructions, as the puncture is performed with breath held in the inspiratory position [1]. There is also an increased risk of severe bleeding complications in patients with chronic renal impairment [1,3,46,60], especially if the kidneys are already reduced in size. The probability that the biopsy can provide therapy-deciding information in this situation is low. Therefore, the size of the kidney should be determined by ultrasound beforehand, and, if possible, it should be ascertained whether it has decreased over the years. The presence of small hyperechoic kidneys is generally indicative of irreversible advanced chronic kidney disease [2]. Percutaneous biopsy of a solitary kidney was long considered an absolute contraindication. Thanks to significant technical improvements that allow percutaneous biopsy to be performed under high-resolution real-time ultrasound, it is now possible to dispense with the open surgical procedure under visual control that was previously common for solitary kidneys [1,61,62]. Notably, today, transplanted kidneys are usually single kidneys that are biopsied with a high degree of safety [63,64].

Studies on the influence of age are partly contradictory. Both younger and older ages have been associated with increased complication rates after percutaneous biopsies [46,58,65,66], but this has not been confirmed in meta-analyses or registry-based studies [3]. Advanced age is definitely not a contraindication to renal biopsies. Recent studies suggest that patients over 60 years of age can safely undergo a renal biopsy [67,68]. Of note, one-third of biopsies for acute kidney injury in this age group have pauci-immune glomerulonephritis [69], which is potentially treatable and has implications for extrarenal organ management. In selected cases, the very elderly beyond the age of 80 years may also benefit from histology-based renal diagnostics in terms of therapy and prognostic assessment [70]. A retrospective case series reported lower rates of end-stage renal failure at one and two years and lower two-year mortality in very old patients with biopsy-proven ANCA-associated vasculitis who were treated compared with those who were not [71].

In general, there is no indication for renal biopsy in cases of multiple bilateral cysts, hydronephrosis, and renal or perirenal infections. By its very nature, percutaneous biopsy is contraindicated when there is a skin infection over the biopsy area. Due to the increased risk of complications, percutaneous access should be avoided under certain circumstances in the case of anatomical abnormalities (Table 2) [1,2].

## 4. Performing the Biopsy

Table 3 summarizes the evidence-based recommendations for performing percutaneous renal biopsy, including precautions to be taken to minimize complications. After explaining the benefits and possible risks of the diagnostic kidney biopsy, the patient’s written informed consent must be obtained 24 h before the procedure. The puncture of the native kidney is carried out in the prone position, with a firm pillow placed under the upper abdomen to support a slight flexion of the trunk. The left kidney is usually easier to visualize on ultrasound and is therefore preferred. Beforehand, the skin and the puncture canal are anesthetized with 1% lidocaine. Real-time sonography has been shown to increase diagnostic yield and reduce biopsy complications [72,73]. Under ultrasound guidance, the tip of the biopsy needle is preferably placed at the lower pole of the kidney, where the likelihood of hitting a major blood vessel is relatively low. Special biopsy transducers allow the angle of the stabbing direction to be precisely aligned with the sonic plane so that the tip of the biopsy needle can be accurately located. In rare cases, when the kidney cannot be adequately visualized by ultrasound, e.g., in cases of extreme obesity, or in instances of complicated anatomy, e.g., bilateral cysts, the biopsy can alternatively be performed CT-guided [74,75]. CT guidance may allow a more precise biopsy needle placement in individual cases and has been shown to provide a high diagnostic yield in patients previously rejected for an ultrasound-guided biopsy [76].

Previous studies indicated as early as the 1990s that spring-operated biopsy devices were able to obtain a greater number of glomeruli per biopsy specimen for light microscopy compared to hand-operated biopsy needles without an increase in bleeding complications [77,78]. Thus, the use of semi-automatic, spring-loaded biopsy devices is now the standard procedure. The choice of the needle size used also depends on the preference of the investigator performing the biopsy. With regard to safety, several studies noted a higher complication rate with 14-gauge (G) needles as compared to 18 G needles. However, the comparison also took place in the context of different biopsy techniques, as with the thinner needles, a spring-loaded biopsy device was used, whereas the 14 G-needles were manually operated [78,79,80]. A more recent prospective study that compared 14 G and 16 G needles using a semi-automatic biopsy device found no difference in the number of glomeruli obtained and the rate of bleeding complications [46]. These findings were basically confirmed for biopsies from transplanted kidneys in a prospective randomized study, although the use of the larger 14 G needle was associated with more pain [81]. However, the meta-analysis by Corapi et al. reported a higher transfusion rate of 2,1% with 14 G needles as compared to 16 G (0.4%) and 18 G needles (0.6%) [3]. On the other hand, recent data suggest that biopsy needles smaller than 16 G may limit the pathologic diagnosis because the sample size is too small and too few glomeruli and blood vessels are being obtained [82,83,84]. Taken together, the use of a 16 G needle seems to be the most reasonable compromise between diagnostic benefit and possible complication rate [1,81]. Needles smaller than 16 G should no longer be used, as the diagnostic yield is often too small.

## 5. Complications

The data on complications can be considered extremely robust and are based on well over 100,000 recorded renal biopsies performed worldwide. The complication rates summarized in this review are derived from meta-analyses of biopsies of native kidneys [3,4,23] and transplanted kidneys [63,64], registry data [58,66], and also representative larger series [46,47,49,54,60]. Bleeding is the most common complication after renal biopsy (Table 4). The risk of bleeding presumably also depends—in addition to the factors already mentioned—on the experience and skill of the physician performing the biopsy. This idea is supported by the Norwegian Renal Biopsy Register, indicating an increased postprocedural risk in smaller centers with <30 biopsies per year [66].

Bleeding can drain into the urinary system, be accompanied by gross hematuria and possibly obstructive symptoms, including bladder tamponade, or it can lead to the formation of a mostly painful renal capsular hematoma, which can spill into the retroperitoneal space, not uncommon with a significant drop in hematocrit and a subsequent need for transfusion. Minor bleeding complications after renal biopsy occur with the following frequency: Microhematuria in almost all patients, macrohematuria in 2–16% of cases, and a drop of hemoglobin by 1 g/dL from the baseline in up to 50% of cases [47]. However, according to more recent data, less than 2% of patients are affected by bleeding requiring red blood cell transfusion. Perinephric hematomas are likely underrepresented in meta-analyses and registry-based evaluations because reported rates are highly dependent on how intensively hematomas were searched for with imaging, particularly if the course was uneventful. For example, two smaller single-center studies from Italy, which prospectively recorded bleeding complications and systematically assessed the incidence of perinephric hematomas with ultrasound also in uncomplicated cases, found an incidence as high as 30–33% [46,51]. Ongoing bleeding due to injury of intrarenal vessels can now be stopped with organ-preserving radiological intervention in most cases [85,86,87,88], and surgical hemostasis is rarely necessary [89,90]. Nowadays, the rate for a required nephrectomy is at 0.01–0.2%.

Mortality attributable to percutaneous renal biopsy was estimated at 0.03% to 0.06% in the large meta-analysis by Poggio et al. [4] based on 87 individual manuscripts on this topic. This would be equivalent to one death per 1600 to 3300 procedures. The estimate is in marked contrast to a nationwide survey from the US that systematically analyzed all hospital admissions, including non-elective admissions, between 2008 and 2012, whenever the International Classification of Disease Revision 9 (ICD-9) procedure code for percutaneous renal biopsy was used. This study, which was included in the meta-analysis by Poggio et al. [4], reported a biopsy-associated mortality of 1.8% [91]. The average length of stay was 10.7 hospital days. A limitation is that it was impossible to clearly attribute deaths occurring during hospitalizations to renal biopsy because the data acquisition was based on codes that were classified into diagnoses-related groups (DRGs) for reimbursements. Another more recent nationwide study from France analyzing more than 55,000 percutaneous biopsies between 2010 and 2019 also suggested a nonnegligible mortality risk, which was estimated to be approximately 1% [92]. However, a substantial number of patients had severe concomitant diseases such as heart failure (10.7%), liver disease (6.1%), history of cancer (24.1%), and acute renal failure (30.3%). These were also significantly associated with death at day 30 after biopsy in univariate and multivariate analyses, independent from major bleeding complications. While the studies by Al Turk et al. [91] and Halimi et al. [92] probably overestimated the mortality risk attributable to biopsy, it is also clear that any biopsy must be weighed against its benefit on a case-by-case basis, and concomitant diseases must also be considered.

Arteriovenous (AV) fistulas are another complication after renal biopsy described in 0.5–10% of cases [2]. Most post-puncture AV fistulas are inapparent and resolve spontaneously. Hemodynamically relevant fistulas, which cause hematuria or can even be associated with a drop in blood pressure and high-output cardiac failure, are very rare and can usually be closed with a radiological intervention [87,93,94]. Other complications include persistent pain lasting longer than 12 h after biopsy (2.5–4.3%) [4,46], but usually remitting spontaneously. Biopsy-related infections in the perirenal soft tissue or accidental punctures of neighboring organs like the liver, spleen, or pancreas are considered an absolute rarity.

## 6. Follow-Up after Biopsy

After the procedure, patients should be on bed rest, and a sandbag is placed under (own kidney) or on top of the kidney (transplanted kidney). Pulse and blood pressure are measured regularly to monitor the circulation. The target blood pressure should be in the normotensive range, below 140/90 mmHg (Table 3). A low normal blood pressure seems to be associated with fewer complications after the biopsy [60]. After 6 h and the following morning, hemoglobin and hematocrit are checked. We also routinely perform ultrasonography of the kidney 1–2 h after the procedure. Large post-biopsy hematomas > 3 cm thick on ultrasound were associated with severe complications in a previous study in 1994 [95]. More recent studies do not demonstrate that a post-puncture hematoma (≤2 cm) within the first hour will predict a complicated course [96]. In contrast, the complete absence of a hematoma on ultrasound achieves a negative predictive value of 95% [97].

**Table 3:** Procedure for percutaneous renal biopsy.

Medical indication	According to Table 1
Consideration of contraindications	According to Table 2
Preparation of the biopsy	
Discontinuation of anticoagulant drugs	
Phenprocoumon/warfarin	5 days before biopsy [1]
Direct oral factor Xa Inhibitors (DOACs)	72 h before biopsy [2]
i.v. heparin for bridging anticoagulation ^a^	to be stopped 6 h before biopsy [1]
Discontinuation of platelet aggregation inhibitors	
Including NSAR	7–10 days before biopsy [1,2]
Low-dose aspirin (100 meg)	Can be continued in case of urgent indication [53,54,55]
Written informed consent	To be obtained at least 24 h before biopsy
Blood pressure (also with medication)	<140/90 mmHg [1,2,3,59,60]
Coagulation Tests	
Thrombocyte count	>120 × 103/µL [1,2]
INR	Must be normal [1,2]
PTT	Must be normal [46]
Bleeding time	Significance controversial [48,49,50]
Administration of desmopressin	Controversial/efficacy not proven [51,52]
Performing the Biopsy	
Positioning of the patient	
Native kidney	Prone with firm pillow under abdomen
Transplanted kidney	Supine position
Pain management	Local anesthesia of the puncture canal
Guidance	Real-time ultrasound [73]
Biopsy device	Semi-automatic spring operated [72,77,78,79]
Needle size	16-gauge preferred [1,3,46,81,82,83,84]
Follow-up after the Biopsy	
Bedrest	According to good clinical practice
Placement of sandbag onto biopsy site	According to good clinical practice
Monitoring of circulation (pulse/blood pressure)	Low normal BP beneficial (120/80 mmHg) [60]
Urine monitoring (mico-/macroscopic hematuria)	According to good clinical practice
Monitoring of hemoglobin	
Routinely 4–6 h after biopsy	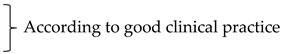
Before discharge after native kidney biopsy	
As needed	
Ultrasonography after 1–2 h	Negative predictive value for bleeding (95%) [96]
In-hospital observation with uncomplicated course	
Native kidney	24 h [47,59,60]
Transplanted kidney	4–6 h [63]
Resuming anticoagulation after biopsy	Preferably not earlier than 48–72 h [1,2]
Management bleeding complications	
Symptomatic hemoglobin drop	Administration of red blood cells as needed
Gross hematuria with obstructive symptoms/bladder	Insertion of Foley catheter and bladder irrigation if needed [2]
tamponade (rare)	
Injury of intrarenal vessels	Selective transcatheter embolization [85,86,87,88,89,90,93,94] Predominantly successful [85,89,90]


^a^ Consider laparoscopic or transjugular approach in patients with mechanical valve replacements or at high risk of thromboembolism.

**Table 4:** Complications after percutaneous renal biopsy.

Author/Year	Data Base/Kind of Study	No. of Procedures	Perinephric Hematoma	Macroscopic Hematuria	Major Complications ^a^	Transfusion Required	Intervention Required ^b,c^	Organ Loss	Death
Poggio [4] 2020	Meta-analysis	118,064	11.0%	3.5%	n.a.	1.6%	0.3%	n.a.	0.06%
Varnell [23] 2019	Meta-analysis	5504 ch.	11–18%	n.a.	n.a.	0.9%	0.7%	n.a.	n.a.
Peters [58] 2019	Registry	2835	2.2%	n.a.	5.6%	1.3%	0.1%	0	0
Lees [54] 2017	Single center	2563	n.a.	n.a.	4,5%	1.8%	0.4%	0	0.4%
Prasad [60] 2015	Single center ^d^	1848 ad. 290 ch.	1.3%	4.7%	5.1%	0.6%	0.5%	0	0
Corapi [3] 2012	Meta-analysis	9474	11–17%	3.5%	1,9%	0.9%	0.6%	0.01%	0.02%
Tøndel [66] 2010	Registry	8573 ad. 715 ch.	3.9–8.1%	1.9%	2.6%	0.9%	0.2%	n.a.	n.a.
Stratta [49] 2007	Single center	1387	7.8%	16.4%	n.a.	0.9%	0.4%	0.07%	0
Manno [46] 2004	Single center prospective	471	33.3%	0.4%	1.2%	0.4%	0.6%	0.2%	0
Whittier [47] 2004	Single center	750	4.0%	4.7%	6.4%	5%	0.7%	0	0.1%
Ho [64] 2022	Meta-analysis Tx kidney	40,082	1.6%	3.2%	0.9%	0.3%	0.2% ^b^ 0.1% ^c^	0.02%	0.01%
Furness [63] 2003	Multi-center Tx kidney control biopsies	2127	2.6%	1.9%	0.4%	0.1%	0.1%	0.05%	0

Abbreviations: Tx kidney: transplanted kidney, ad.: adults, ch.: children, n.a.: not analyzed. ^a^ Defined by the requirement of either transfusion or radiological, surgical, or urological procedure or otherwise intervention. ^b^ radiologic embolization, ^c^ surgical hemostasis, and ^d^ including 361 transplant biopsies.

The majority of bleeding complications following biopsy of a native kidney manifest within 6–8 h. However, if the observation time is less than 12 h, 10–15% of bleeding escapes timely detection and possible treatment [47,60]. Therefore, it is reasonable to discharge patients after a biopsy of a native kidney only after 24 h of clinical monitoring. Severe bleeding complications occur only in exceptional cases thereafter. In contrast, in the study by Furness et al. [63]. which included 2167 protocol biopsies after renal transplantation, all rare relevant bleeding complications were observed within the first 4 h after biopsy. Therefore, elective patients after transplant kidney biopsy with an uncomplicated course during an observation period of 4–6 h can mostly be discharged on the same day. Overall, complications appear to occur less frequently after transplant kidney biopsies than after biopsies of native kidneys, presumably because of the easier access (Table 4).

## 7. Alternative Techniques

If a bleeding tendency cannot be corrected and there is an urgent indication to perform a biopsy, alternative techniques may be used. Surgical procedures, open or laparoscopic renal biopsy, or radiology interventional access via the internal jugular vein (TJRB) may be considered. The advantages of the surgical approach are that the tissue sample is taken under visual control, and any bleeding can be sutured under optimal conditions. Disadvantages include the need for general anesthesia and a prolonged hospital stay associated with the surgical approach. Several case series published to date have shown that the laparoscopic biopsy technique can obtain sufficient material for histologic diagnosis with an overall low complication rate [98,99,100,101].

In TJRB, a special catheter is advanced into a peripheral interlobar vein via the renal vein so that the tip comes to rest in a wedge position. The aspiration biopsy is then performed in the direction of the renal capsule, whereby this must not be perforated. Any parenchymal hemorrhage that may occur drains through the renal venous system under these conditions so that there is no blood loss to the outside. In cases where accidental capsular perforation is identified, the bleeding can usually be stopped in the same session by selective coil embolization [102]. The procedure has been shown to be safe to use in the hands of an experienced interventional radiologist in high-risk patients with uncorrectable coagulopathy capable of obtaining sufficient biopsy material. Cluzel et al. retrospectively analyzed a series of 400 TJRB and compared them with 400 percutaneous biopsies during the same observation period [103]. Sufficient renal tissue for histological diagnosis was obtained in 95.8% and 95.5% of cases, respectively, with both procedures. The number of contained glomeruli per biopsy was only slightly lower with TJRB (9.8 ± 7.6 versus 11.2 ± 7.7), and serious complications occurred with equal frequency (1.0% versus 0.8%). Another series of 39 high-risk patients for bleeding complications from the Mayo Clinic basically confirmed these results. Twenty-four patients (63%) had a platelet count <75 × 10^9^/L and eleven (29%) had an INR > 1.4. TJRB achieved adequate diagnostic yield in 97% of cases, and only one patient (2.6%) experienced a major bleeding complication [104]. A recent systematic review of 17 published studies involving 1321 biopsies concluded that TJRB is a feasible procedure for obtaining renal tissue for diagnosis, with an acceptable risk of severe bleeding in patients excluded from percutaneous biopsy because of a coagulation disorder [105]. Consistent with this, the aforementioned nationwide cohort study from France by Halimi et al. [92] comparing the transjugular (5305 patients) with the percutaneous route (55,026 patients) found a lower risk of major bleeding with TJRB after accounting for bleeding risk factors (OR 0.88; 95% CI 0.78–0.99). Another advantage of TJRB is that liver and kidney tissue can be obtained through the same access when indicated [106]. The principal disadvantage is the need for X-ray contrast agent application. The occurrence of renal failure induced by contrast medium in connection with TJRB was reported in individual cases [104]. The advantages and limitations of the various biopsy techniques and special indications are shown in Table 5.

## 8. Conclusions

Renal biopsy is the method of choice for the diagnosis of parenchymal kidney disease and allows differentiation of active inflammation from irreversible chronic changes with corresponding implications for prognosis and possible treatment options. In the majority of cases, the information obtained from the biopsy has a decisive influence on optimized therapy. With the use of semi-automated spring-loaded biopsy devices and high-resolution real-time ultrasound, the percutaneous kidney biopsy is now a standard procedure that is safely performed. Major bleeding complications manifest within the first 12–24 h but are overall rare and can usually be controlled by radiology intervention. Absolute contraindications to the percutaneous route are coagulation disorders and medically uncontrolled hypertension. In addition, anemia and a more severe renal function impairment at the time of biopsy are associated with an increased risk of bleeding. If pre-existing risks cannot be adequately reduced and there is an urgent indication for biopsy, alternative procedures, such as a laparoscopic or a transjugular approach, should be considered. The use of a 16 G needle provides the optimum compromise of post-procedural complication and a sufficient tissue yield for the histological diagnosis. It is standard today to examine the native kidney biopsy with light microscopy, immunohistochemistry, and electron microscopy. Future implementation of new modern pathology techniques on individual biopsies, such as multiplex immunofluorescence, spatial proteomics, and transcriptomics [107,108,109], will add to diagnostic capabilities at the molecular level. It is hoped that along with this, a more accurate and comprehensive understanding of the development and progression of kidney disease will also lead to a refinement of treatments.

## Figures and Tables

**Table 2:** Contraindications to percutaneous renal biopsy.

Clotting disorder [1,2,46]
Thrombocytopenia (platelet count <120 × 10^3^/µL) [1,2]
Medication uncontrolled hypertension (>140/90 mmHg) [1,2,3,49,59,60]
Small hyperechoic kidneys on ultrasound [1,2]
Patient’s inability to follow instructions [1,2]
Patient’s inability to provide informed consent [1,2]
^a^ Anatomic anomaly/horseshoe kidney [2]
Multiple bilateral cysts [2]
Hydronephrosis [1,2]
Active kidney infection/pyelonephritis or perirenal abscess [1,2]
Skin infection at the site of needle insertion [2]

^a^ In case of an urgent indication, a transjugular approach should be sought [2].

**Table 5:** Comparison of the different biopsy procedures.

Access Route	Clinical Situation	Main Advantage	Limitation
Percutaneous ultrasound-guided biopsy (PUSB)	Normal bleeding risk Given indication Consideration of contraindications	Little technical effort	Not always practicable
Percutaneous CT-guided biopsy	Like in PUSB, but kidney cannot be adequately visualized on ultrasound [75,76] Extreme obesity [75,76] Anatomic abnormality (e.g., cysts)	More precise placement of biopsy needle [76]	Higher technical effort Radiation exposure
Transjugular renal biopsy (TJRB)	Increased bleeding risk Urgent indication Coagulation disorder [102,104,105,106] Anatomic abnormality [102]	Avoidance of blood loss through the renal capsule [102] Simultaneous liver biopsy via the same access if indicated [103,106]	Skilled interventional radiologist required Radiation exposure Contrast media exposure
Surgical/laparoscopic approach	Like in TJRB [98,100,101]	Sampling and hemostasis under visual control	General anesthesia required Operating theatre required Prolonged hospitalization
